# *MBL2* Genotypes and Their Associations with MBL Levels and NICU Morbidity in a Cohort of Greek Neonates

**DOI:** 10.1155/2015/478412

**Published:** 2015-03-24

**Authors:** Matthaios Speletas, Antonios Gounaris, Eirini Sevdali, Maria Kompoti, Katerina Konstantinidi, Rozeta Sokou, Elena Tsitsami, Anastasios E. Germenis

**Affiliations:** ^1^Department of Immunology & Histocompatibility, School of Health Sciences, Faculty of Medicine, University Hospital of Larissa, University of Thessaly, Biopolis 3, 41500 Larissa, Greece; ^2^Neonatal Intensive Care Unit, School of Health Sciences, Faculty of Medicine, University Hospital of Larissa, University of Thessaly, Biopolis 3, 41500 Larissa, Greece; ^3^Neonatal Intensive Care Unit, General Hospital of Nikea “Agios Panteleimon”, 11634 Athens, Greece; ^4^Intensive Care Unit, Thriassion General Hospital of Eleusis, 19600 Athens, Greece

## Abstract

The objective of this study was to assess the frequency of *MBL2* genotypes and their associations with MBL levels and various morbidities of a neonatal intensive care unit (NICU). One hundred and thirty-four (134) NICU (83 term and 51 preterm) and 150 healthy neonates were enrolled in the study. *MBL2* genotype and MBL serum levels at birth were determined prospectively by PCR-RFLP-sequencing and enzyme-linked immunosorbent assay, respectively. NICU neonates displayed significantly lower MBL serum levels compared to healthy ones. MBL deficiency, defined as the low *MBL2* expression group (XA/O and O/O), was significantly associated with an increased risk of respiratory morbidity, especially transient tachypnea of the newborn and respiratory distress syndrome (RDS). Moreover, an increase of 100 ng/mL of serum MBL levels decreases by 5% the risk of total respiratory morbidity and by 7% the risk of RDS, after correction for prematurity and sex and regardless of the presence of infections. Our study further supports the notion that neonates with MBL deficiency and low MBL serum levels at birth may be at higher risk of developing severe respiratory complications.

## 1. Introduction

Nowadays, it is well recognized that the neonatal immune system relies largely on the function of innate immunity. Monocytes, granulocytes, mast cells, natural killer cells, and other innate immune system mediators, including complement factors and acute phase proteins, are critically important to prevent infections [1]. The interaction between individual genetic factors and the proinflammatory environment may determine the newborns' “immune phenotype,” affecting their predisposition to infections [1, 2]. Genetic epidemiological studies identified that allelic variations of genes encoding cytokines, such as interleukin-6 (IL-6) and tumor necrosis factor-alpha (TNF-*α*), were associated with perinatal infections [3, 4]. In this context, recent studies have also reported that polymorphisms of the gene encoding mannose-binding lectin (*MBL2*) and/or low MBL serum levels might be associated with perinatal infections and/or preterm delivery [5–12].

MBL is an acute phase protein that plays a key role in the first line immune responses as component of neonate innate immunity, since the adaptive immunity arms are not sufficiently developed. MBL belongs to the collectin family of proteins, which also includes lung surfactant protein-A (SP-A) and SP-D [13]. It bounds to mannose or sugar motifs, which are present in a wide range of microorganisms, and is able to activate the complement system in an antibody and C1-independent manner [13, 14]. MBL may also interact directly with cell surface receptors and thereby promote opsonophagocytosis by a complement-independent pathway [13, 15, 16].

Although MBL serum concentrations show important changes with age [17], they are primarily determined by polymorphisms in both exon 1 and promoter of the* MBL2* gene [13, 18]. Three single nucleotide polymorphisms (SNPs) at codons 52, 54, and 57 of exon 1 are frequently referred to as variants D (rs5030737), B (rs1800450), and C (rs1800451), respectively, while the wild-type allele is referred to as allele A and the O allele represents the variant alleles D, B, or C [13, 18]. It has been proposed that the presence of the O allele impairs the oligomerization of MBL, resulting in reduced levels of functional protein circulating in the serum [19]. Additionally, SNPs in the promoter region at positions -550 and -221, known as variants H/L (rs11003125) and X/Y (rs7096206), respectively, also influence* MBL2* expression, although only the X variant significantly reduces MBL serum levels [13, 19]. Subsequently, the combination of the genetic alterations into both exon 1 and promoter results in 3 MBL genotype expression groups, which are associated with high (YA/YA, YA/XA), medium (XA/XA, YA/O), and low (XA/O, O/O) MBL serum levels [5, 13, 19].

MBL deficiency, characterized by low MBL levels and/or the low expression genotype (XA/O or O/O) [5, 19], has been associated with a decreased ability of opsonization of microorganisms and an increased susceptibility to infections, mainly in early childhood and in immunocompromised individuals [20–22]. Considering both the interest on the role of MBL in the neonates' defense against infections and the presence of inconclusive results in the literature about the clinical significance of MBL deficiency in newborns [5–12], this study was scheduled in order to clarify the association of both functional and genotypic MBL deficiency with the causes of neonatal morbidity in a cohort of Greek NICU neonates.

## 2. Methods

### 2.1. Study Population

One hundred and thirty-four neonates (93 term and 51 preterm) admitted to the NICU and 150 healthy neonates from the Department of Neonatology of the General Hospital of Nikea “Agios Panteleimon,” Athens, Greece, were enrolled in the study, after obtained informed consent from their parents. Blood was obtained from the neonates (and never from the umbilical cord) usually 2-3 h, and always within 12 h, after birth. In cases of a suspected infection, a comprehensive laboratory examination was performed, including blood and urine cultures, leukocyte counts, and C-reactive protein (CRP) levels. The diagnosis of all aspects of respiratory morbidity, including respiratory distress syndrome (RDS), was based on standard definition criteria [23]. In all cases, a detailed mother's medical history was also obtained. Demographic and clinical data of neonates included in this study are summarized in Supplementary Table 1 (see Supplementary Material available online at http://dx.doi.org/10.1155/2015/478412). The study protocol was approved by the local medical ethics committee.

### 2.2. Molecular Techniques

Genomic DNA was extracted from whole blood using QIAmp DNA Blood Mini Kit (Qiagen, UK) according to manufacturer's instructions. The detection of* MBL2* alterations was performed by allele-specific polymerase chain reaction followed by restriction fragment length polymorphism (PCR-RFLP) analysis as described [24], with some modifications. In brief, the forward primers, in both reactions, were modified at the 3′ end, creating restriction enzyme recognition sites (*MwoI* for the* MBL2*-Arg52Cys polymorphism [rs5030737] and* DrdI* for the* MBL2* -550G>C [rs11003125]), so that if a polymorphism is present, PCR-RFLP analysis will create digestion fragments, visible on agarose gels. The detection of the other* MBL2* polymorphisms was based on the fact that the polymorphisms result in the creation of a DNA sequence recognized by the restriction enzymes* BanI* (for the detection of Gly54Asp, rs1800450),* MboII* (for the Gly57Glu, rs1800451), and* BtgI* (for the -221G>C, rs7096206). The primer pairs, the conditions of PCR, and the expected fragments of RFLP are presented in detail in Table 1.

For the confirmation of results, about half of all PCR products were also purified by Qiagen PCR Purification System (Qiagen) and directly sequenced using an ABI Prism 310 Genetic Analyzer (Applied Biosystems, Foster City, CA) and a Big Dye Terminator DNA sequencing kit (Applied Biosystems).

### 2.3. Measurement of MBL Levels

The part of blood sample for the measurement of MBL levels was collected in a covered test tube without anticoagulant. Fifteen to thirty minutes after collection, the blood samples were centrifuged at 2,000 ×g for 10 min at room temperature and serum samples were stored at −80°C until analysis. The oligomerized MBL levels were measured using an immunoassay (MBL Oligomer ELISA, Antibody Shop, Copenhagen, Denmark), according to manufacturer's instructions. The linear range was 0–40 *μ*g/mL. Sera were diluted 1 : 100 in the sample diluent provided by the manufacturer. The lowest detectable MBL concentration was 10 ng/mL. For subsequent statistical analysis, results below the limit of detection were allocated a value of 10 ng/mL.

### 2.4. Statistical Analysis

Categorical variables were analyzed with Fisher's exact test. Normality of continuous variables was assessed with Kolmogorov-Smirnov test. Normally distributed data were analyzed with Student's *t*-test and one-way ANOVA, as appropriate. Skewed data were analyzed with nonparametric methods (Mann-Whitney or Kruskal-Wallis test, as appropriate). Associations of continuous end-point variables were assessed by linear regression, as appropriate. Binary variables were entered as dependent in univariable and multiple logistic regression models, as appropriate. The predictive ability of fitted logistic models was assessed with receiver operating curve (ROC) analysis. For all analyses, alpha was set at 0.05 (2-sided). Data analysis was performed with SPSS 17.0 (IBM Corporation, NY, 2008).

## 3. Results

### 3.1. *MBL2* Genotypes/Haplotypes, Genotypic MBL Deficiency, and Neonatal Morbidity

**Figure 1:**
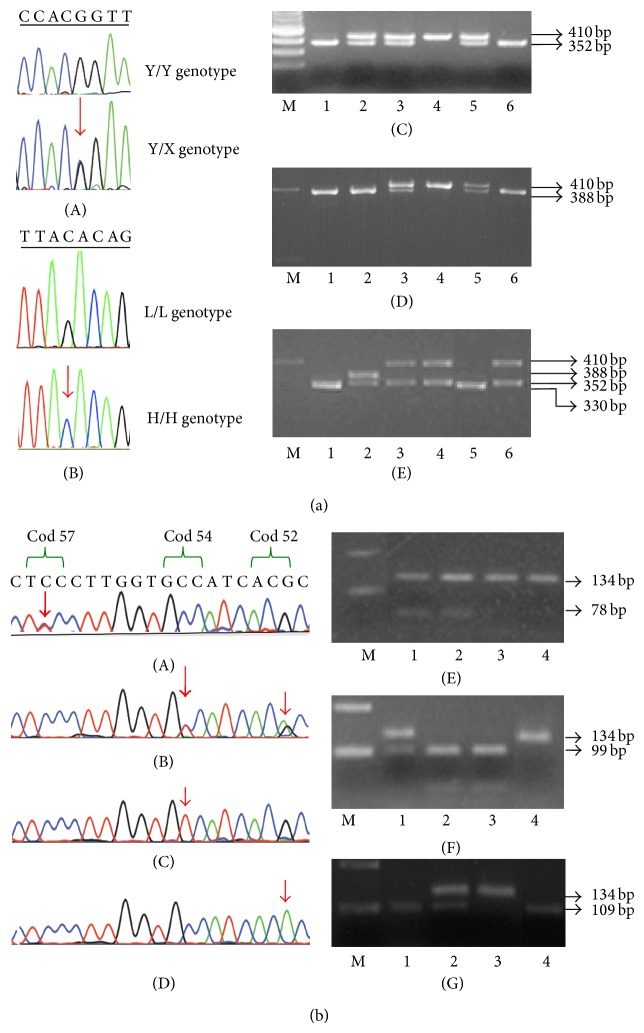
The detected polymorphisms of* MBL2* gene in Greek neonates. (a) Detection of promoter polymorphisms. Sequencing analyses indicating the presence of (A) the X/Y (-221G>C) and (B) the L/H (-550G>C) polymorphisms. (C) Representative digestion showing the X/Y polymorphism. M: 100 bp ladder molecular weight marker (New England Biolabs, UK). Lanes 1 and 6: homozygotes for the Y allele, lane 4: homozygotes for the X allele, and lanes 2, 3, and 5: samples carrying both X and Y alleles. (D) Representative digestion showing the L/H polymorphism. M: 200 bp ladder molecular weight marker (Invitrogen, UK). Lanes 1, 2, and 6: homozygotes for the L allele, lane 4: homozygotes for the H allele, and lanes 3 and 5: samples carrying both L and H alleles. (E) Representative digestion using a mixture of both restriction enzymes* BtgI* and* DrdI* for the detection of promoter polymorphisms. M: 200 bp ladder molecular weight marker. Lanes 1 and 5: samples with the LY haplotype, lane 2: sample with the HY/LX genotype, and lanes: 3, 4, and 6: samples with the HX/HY genotype. (b) Detection of exon 1 polymorphisms. Sequencing analyses of samples indicated (A) a sample heterozygous for the variant allele of Gly57Glu (rs1800451) polymorphism, (B) a sample double heterozygous for the variant alleles of Arg52Cys (rs5030737) and Gly54Asp (rs1800450) polymorphisms, (C) a sample homozygous for the variant allele of Gly54Asp polymorphism, and (D) a sample homozygous for the variant allele of Arg52Cys polymorphism. (E) Representative digestion showing the presence of the Gly57Glu polymorphism. M: 200-bp ladder molecular weight marker. Lanes 1 and 2: heterozygotes, lanes 3 and 4: wild-type (wt) samples. (F) Representative digestion showing the presence of the Gly54Asp polymorphism. M: 200-bp ladder molecular weight marker. Lane 1: heterozygous sample, lanes 2 and 3: wt samples, and lane 4: homozygous sample for the variant allele. (G) Representative digestion showing the presence of the Arg52Cys polymorphism. M: 200-bp ladder molecular weight marker. Lanes 1 and 4: wt samples, lane 2: heterozygous sample, and lane 3: homozygous sample for the variant allele. All PCR and RFLP samples were run on 2% agarose gel, and the fragments with size lower than 60-bp were not visible.

Healthy and NICU neonates, as well as the NICU subgroups (term and preterm), were classified on the basis of the combined genotypes of promoter and exon 1 polymorphisms, in six groups (YA/YA, YA/XA, XA/XA, YA/O, XA/O, and O/O). Subsequently, a further classification in three groups was performed according to MBL expression levels, namely, high (YA/YA, YA/XA), medium (XA/XA, YA/O), and low (XA/O, O/O) [5, 13, 19]. Moreover, we were able to assign the six commonly found haplotypes (HYA, LYA, LXA, HYD, LYB, and LYC) of* MBL2* gene. The rs7095891 SNP in the 5′ untranslated region at position +4 (P/Q) was not detected and, hence, we did not discriminate between the common LYPA and LYQA haplotypes [6, 19]. Examples of the detection of* MBL2* gene alterations are presented in Figure 1.

The observed genotype frequencies of* MBL2* gene along with the respective MBL serum levels are presented in Table 2. Genotypic MBL deficiency (XA/O or O/O genotypes) was detected in 17.3% of healthy and 21.6% of NICU neonates. Compared to healthy and term NICU neonates, the preterm NICU neonates displayed genotypic MBL deficiency in a higher, although not significant, frequency (25.5% versus 17.3% and 19.3%, resp.). Comparing the individual frequencies of the above mentioned six common haplotypes and the subsequent* MBL2* genotypes between healthy and NICU neonates (Table 3), no significant differences were found.

The most common* MBL2* polymorphism into exon 1 was that of codon 54 (B allele) and there was no significant difference in its prevalence between healthy and NICU newborns (allele frequencies 19.0% and 20.5%, resp.; *P* = 0.709). Considering the rs5030737 SNP (codon 52, D allele), no healthy individual was homozygous, while 5 out of 20 healthy heterozygotes carried also the B allele (allele frequency: 6.7%); in NICU group 2 neonates were homozygotes and 13 heterozygotes (3 of them carried also the B allele; allele frequency 6.3%, *P* = 0.417). Moreover, only 2 NICU neonates carried the C allele (rs1800451, codon 57) in heterozygous state, in contrast to nonhealthy newborn (allele frequencies 0.7% versus 0%, *P* = 0.135). At the end, no significant differences considering the B, C, and D alleles were observed between term and preterm neonates into NICU group (21.7% versus 18.6%, *P* = 0.624; 1.2% versus 0.0%, *P* = 0.269; 4.2% versus 9.6%, *P* = 0.089, resp.).

The presence of genotypic MBL deficiency was significantly associated with an increased probability of respiratory morbidity (*P* = 0.039) and especially with transient tachypnea of the newborn (TTN, *P* = 0.006). Interestingly, no significant associations between genotypic MBL deficiency and perinatal infections and/or sepsis were observed (*P* > 0.05 in all cases).

### 3.2. MBL Serum Levels and Neonatal Morbidity

**Figure 2:**
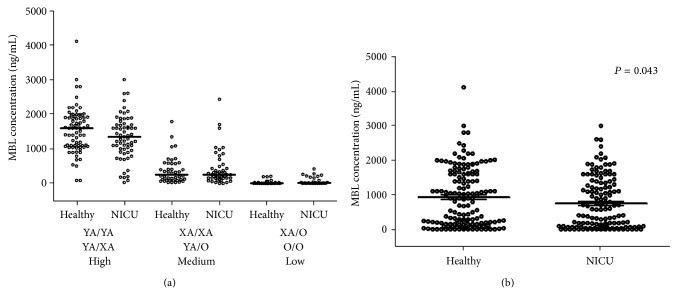
(a) Scatterplot of MBL serum levels in the neonates of the study, according to the high, medium, and low* MBL2* genotype expression groups (corresponding* MBL2* haplotypes are shown). Median is illustrated. (b) Scatterplot of MBL serum levels in healthy and NICU neonates of the study.

As presented in Table 2 and Figure 2(a), MBL concentration was significantly associated with the three* MBL2* genotype groups both in healthy and NICU neonates. The healthy neonates also displayed significantly higher MBL levels compared to NICU ones (median range: 900 ng/mL, 10–4110 versus 580 ng/mL, 10–3000; Figure 2(b)). Interestingly, the finding of higher MBL levels in healthy neonates is not assigned to prematurity, since no significant difference was observed on MBL levels between preterm and term neonates both in NICU (median range: 340, 10–2200 ng/mL versus 600 ng/mL, 10–3000; *P* = 0.372) and in total (median range: 380, 10–2200 ng/mL versus 715 ng/mL, 10–4110; *P* = 0.110). Additionally, male neonates displayed lower MBL serum levels compared to females, although the difference was not reached to be significant (Supplementary Figure 1); this is attributed to the fact that, in our cohort of neonates, females displayed more frequently the genotypes YA/YA and YA/XA, which are associated with higher MBL serum levels (Supplementary Figure 1).

Considering that there is no consensus on the definition of the functional MBL deficiency, we initially used three different cut-off values of MBL concentration (150 ng/mL, 400 ng/mL, and 700 ng/mL) (Table 2), according to the recent literature data [5, 8]. As expected, a positive strong correlation was observed between* MBL2* low expression genotypes and all cases of functional MBL deficiency (*P* < 0.001). Similarly with the genotypic MBL deficiency, the functional one was significantly associated with the development of respiratory morbidity (*P* = 0.001, *P* = 0.026, and *P* = 0.005, for MBL levels lower than 150 ng/mL, 400 ng/mL, or 700 ng/mL, resp.) and especially with TTN (*P* < 0.001, *P* = 0.018, and *P* = 0.014, resp.).

**Figure 3:**
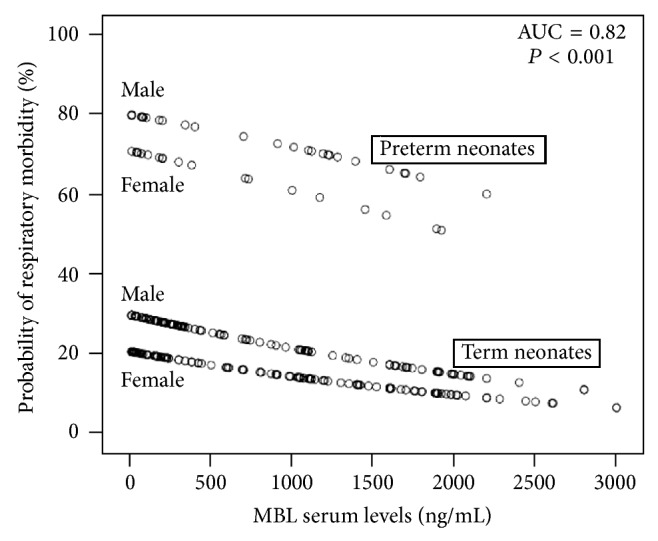
Multivariate logistic regression model indicated the probability of respiratory morbidity according to the MBL serum levels, corrected for prematurity and sex.

In order to further identify the best possible discrimination between the low and high value (cut-off) of MBL levels for the prediction of the development of respiratory morbidity, we performed ROC curve analysis. Interestingly, using MBL serum levels as a single variable, their prognostic value for the development of respiratory morbidity was poor (AUC < 0.7). Taking into account that the development of respiratory morbidity was also significantly affected by the male sex (*P* = 0.018) and the prematurity (*P* < 0.001), we performed a further logistic regression analysis, including MBL levels along with prematurity and sex as independent variables. The predictive value of the emerged model was strong, indicating that an increase of 100 ng/mL of serum MBL levels decreases by 5% the risk of the development of respiratory morbidity (AUC = 0.82, *P* < 0.001; Figure 3). Moreover, premature newborns in NICU displayed a 9.5-fold increased risk for the development of respiratory morbidity compared to term newborns of the same sex and similar MBL serum levels (OR: 9.549, 95% CI: 4.761–19.153). Additionally, male newborns in NICU exhibited a 1.6-fold increased risk for the development of respiratory morbidity compared to females with similar MBL serum levels (OR: 1.641, 95% CI: 0.909–2.961).

Bearing in mind both the fact that MBL serum levels are fundamentally affected by* MBL2* genotypes and the aforementioned results, we further explored whether the specific* MBL2* genotypes affect the risk of respiratory morbidity. Interestingly, we demonstrated that the presence of the medium (XA/XA, YA/O) and the low (XA/O, O/O)* MBL2* genotype groups increased the risk for respiratory morbidity by 2.3- and 3.4-fold, respectively, compared to high (YA/YA and YA/XA)* MBL2* group, after correction for prematurity (OR: 2.301, 95% CI: 1.004–5.273 and OR: 3.429, 95% CI: 1.256–9.395, resp.).

Moreover, we demonstrated that the development of RDS was weakly associated with MBL serum levels (OR: 0.928, 95% CI: 0.864–0.996, *P* = 0.039) and strongly with prematurity (OR: 15.001, 95% CI: 6.014–37.419, *P* < 0.001). Accordingly, a logistic regression model including prematurity and MBL serum levels predicted that an increase of 100 ng/mL of MBL resulted in a 7% decreased risk of RDS development in NICU, after correction for prematurity (AUC = 0.835, *P* < 0.001).

Considering other parameters of neonatal morbidity, a univariate logistic regression analysis indicated that the development of jaundice was also significantly associated with MBL serum levels (*P* = 0.042), along with the male sex (*P* = 0.047) and the prematurity (*P* < 0.001). However, after a multivariate regression analysis, only the prematurity was independently associated with the development of jaundice (adjusted OR: 11.700, 95% CI: 5.700–23.900, *P* < 0.001).

## 4. Discussion

Our results suggest that the presence of genotypic MBL deficiency is significantly associated with respiratory complications in NICU, while any increase of MBL serum levels seems to result in a decrease of the risk of respiratory morbidity and RDS and might have important therapeutic implications.

Previous studies have shown that serum MBL levels are lower in newborns, depending also on the gestational age, and increase during the first weeks after birth [5, 8, 17, 25]. Considering also that there is no consensus about the definition of functional MBL deficiency in newborns, we initially used three different cut-off values of MBL levels for our statistical analyses. However, our data demonstrate that MBL serum levels, as a single variable, could not predict the risk of newborn morbidity. Indeed, the latter is also affected by other variables, as prematurity and sex, suggesting that nonspecific value of serum MBL could be a clinically meaningful cut-off indicative of functional MBL deficiency in newborns.

Neonatal respiratory morbidity, including RDS, TTN, perinatal asphyxia due to fetal distress, congenital pneumonia, air leaks, and persistent pulmonary hypertension, has been attributed to abnormal levels and/or composition of surfactants [26–28]. Additionally, a study performed in adults has identified a positive correlation between MBL deficiency, especially of B allele, and acute respiratory distress syndrome (ARDS), independently of the presence of septic shock or pneumonia [29]. Similarly, our study suggests that MBL deficiency in newborns might contribute to the development of respiratory morbidity, regardless of the presence of infections.

Interestingly, the contribution of MBL deficiency in the pathophysiology of several respiratory diseases has extensively been analyzed in recent studies [30–34]. Thus, Lin and coworkers identified that the genotypic MBL deficiency increases the risk of recurrent infective exacerbations in patients with chronic obstructive pulmonary disease and worsens the disease outcome [30]. Moreover, genotypic and/or functional MBL deficiency has been associated with respiratory disease progression in cystic fibrosis [31], as well as with bronchiectasis formation in common variable immunodeficiency [32, 33]. In this context, Chalmers et al. have suggested that MBL might be an important modifier of disease severity in noncystic fibrosis bronchiectasis, since patients with low-expressing MBL genotypes and/or low MBL serum levels (<200 ng/mL) displayed a higher frequency of chronic colonisation with bacteria, more frequent severe exacerbations during follow-up, and a worsen quality of life [34].

The mechanism by which MBL protects the lungs is unclear, but several mechanisms could be suggested, involving both anti-infectious and anti-inflammatory processes [35, 36]. Moreover, the genes encoding MBL and some surfactant proteins, such as SP-A1, SP-A2, and SP-D, display a high sequence homology and are also located on the long arm of chromosome 10q25, derived from a common ancestor [37]. Consequently, the increased incidence of respiratory morbidity in neonates with MBL deficiency might be due to SNPs of the genes encoding surfactant protein(s), which may be in linkage disequilibrium with* MBL2* ones; obviously, this hypothesis remains to be explored. Another explanation might be a direct effect of MBL in lungs, considering that MBL shares common receptors and activities with SP-A and SP-D, such as the receptors calreticulin, C1qRp, SP-A receptor, and CD91, or the binding and the subsequent cleaning of the lungs by free nucleic acids [38–40]. In this context, it is noteworthy that the administration of purified MBL in a patient with severe cystic fibrosis, displaying also MBL deficiency and severe bronchopulmonary infection, resulted in a dramatic improvement of lung function, along with a stabilization of her clinical condition [36].

In conclusion, the results of this study indicate that low MBL levels and/or genotypic MBL deficiency predispose to respiratory complications in neonates, especially TTN and RDS. Considering that the substitution therapy with MBL is now available and safe, the confirmation of the abovementioned association might provide the rationale for a controlled trial to evaluate the efficacy of early administration of MBL in the management of respiratory complications of NICU neonates with low MBL serum levels and/or genotypic MBL deficiency.

## Supplementary Material

Supplementary Figure 1. A. Scatterplot of MBL serum levels and B. prevalence of MBL2 genotype groups in the neonates of the study, according to their sex. The graph B was prepared by “Mac Statistics Wizard” software (version 10.0.4) ".
Supplementary Table 1. Clinical and demographic characteristics of the study population.

## Figures and Tables

**Table:** (a) PCR primers and conditions

Region	Position^*^	Sequence^#^	Conditions of both reactions	PCR product
Promoter	4421-4446 4808-4830	5′-GAAAATGCTTACCCAG(G)ACAAGCCTGT-3′ 5′-GTCTCCTCATATCCCCAGGC-3′	94°C for 5 min, followed by 30 cycles (94°C for 30 s, 64°C for 30 s, and 72°C for 30 s) and a final elongation at 72°C for 5 min	134 bp
Exon 1	5191-5218 5305-5324	5′-CATCAACGGCTTCCCAGG(C)G(A)CAAGATGGG-3′ 5′-GTCTCCTCATATCCCCAGGC-3′		410 bp

**Table:** (b) Expected fragments of RFLP genotyping for *MBL2* gene

Polymorphic sites	Enzyme	RFLP conditions	Wild-type homozygotes (bp)	Mutant homozygotes (bp)	Wild-type & mutant heterozygotes (bp)
-550G>C (rs11003125)	*DrdI *	4 h at 37°C	410 (H allele)	388 + 22 (L allele)	410 + 388 + 22 (H & L)
-221G>C (rs7096206)	*BtgI *	4 h at 37°C	410 (X allele)	352 + 58 (Y allele)	410 + 352 + 58 (X & Y)
Arg52Cys (rs5030737)	*MwoI *	12 h at 60°C	109 + 25	134	134 + 109 + 25
Gly54Asp	*BanI *	4 h at 37°C	99 + 35	134	134 + 99 + 35
Gly57Glu	*MboII *	4 h at 37°C	134	78 + 56	134 + 78 + 56

**Table:** (c) *MBL2* promoter genotypes: RFLP in *MBL2 *promoter using a combination of both *DrdI* and *BtgI* enzymes

Genotypes	Respective haplotypes^∧^	410 bp	388 bp	352 bp	330 bp
*PCR product *		✓			
HY/HY	HYA/HYA, HYA/HYD, HYD/HYD			✓	
HY/LY	HYA/LYA, HYA/LYB, HYA/LYC, HYD/LYA, HYD/LYB, HYD/LYC			✓	✓
HY/LX	HYA/LXA, HYD/LXA		✓	✓	
LX/LX	LXA/LXA		✓		
LX/LY	LXA/LYA, LXA/LYB, LXA/LYC		✓		✓
LY/LY	LYA/LYA, LYA/LYB, LYA/LYC, LYB/LYB, LYB/LYC, LYC/LYC				✓

^*^ *MBL2* gene numbering is according to GenBank accession number NG_008196.1.

^#^In forward primers of both reactions, the nucleotides in parentheses were modified and changed to the underlined ones.

^∧^The common haplotypes of *MBL2*, identified also in this study, are the HYA, LYA, LXA, HYD, LYB, and LYC.

**Table 2:** Overview of MBL concentrations and *MBL2* genotype.

	Healthy		NICU (ne*ο*natal intensive care unit)							
	(number 150)		Total (number 134)			Term (number 83)		Preterm (number 51)		
	MBL (ng/mL)		MBL (ng/mL)			MBL (ng/mL)		MBL (ng/mL)		
	*n* (%)	Median (range)	*n* (%)	Median (range)	*P*1, *P*2 values^*^	*n* (%)	Median (range)	*n* (%)	Median (range)	*P*3, *P*4 values^#^
*MBL2* genotype										
YA/Y*Α*	50 (33.3)	1725 (100–4110)	42 (31.3)	1515 (100–3000)	0.798 0.082	23 (27.7)	1610 (190–3000)	19 (37.3)	1190 (100–2200)	0.407 **0.033**
YA/ΧA	26 (17.3)	1290 (550–1990)	21 (15.7)	1230 (40–2080)	0.750 0.480	13 (15.7)	1350 (740–2080)	8 (15.7)	1055 (40–1690)	0.998 0.082
ΧA/ΧA	6 (4.0)	885 (700–1600)	9 (6.7)	600 (340–1790)	0.333 0.088	7 (8.4)	600 (420–1090)	2 (3.9)	1065 (340–1790)	0.341 NA
YA/O	42 (28.0)	235 (10–2440)	33 (26.7)	180 (40–1350)	0.623 0.452	24 (28.9)	210 (40–1350)	9 (17.6)	105 (50–710)	0.247 0.384
XA/O	16 (10.7)	45 (10–430)	17 (12.7)	30 (10–210)	0.637 0.065	11 (13.3)	10 (10–210)	6 (11.8)	10 (10–200)	0.825 0.339
O/O	10 (6.7)	10 (10–290)	12 (9.0)	10 (10–200)	0.505 0.473	5 (6.0)	10 (10–10)	7 (13.7)	10 (10–200)	0.169 1.000

*MBL2* genotype groups										
High YA/YA, YA/XA	76 (50.7)	1600 (100–4110)	63 (47.0)	1350 (40–3000)	0.719 0.068	36 (43.4)	1600 (190–3000)	27 (52.9)	1170 (40–2200)	0.521 **0.012**
Medium XA/XA, YA/O	48 (32.0)	260 (10–2440)	42 (31.3)	265 (10–1790)	0.932 0.833	31 (37.3)	330 (40–1350)	11 (21.6)	180 (50–1790)	0.160 0.367
Low XA/O, O/O	26 (17.3)	25 (10–430)	29 (21.6)	10 (10–210)	0.451 **0.044**	16 (19.3)	10 (10–210)	13 (25.5)	10 (10–200)	0.499 0.569

MBL functional deficiency					*P*5 value^∧^					*P*6 value^∧^

MBL levels ≤150 ng/mL	30 (10.0)		42 (31.3)		0.091	24 (28.9)		18 (35.3)		0.578
MBL levels ≤400 ng/mL	61 (40.7)		62 (46.3)		0.550	35 (42.2)		27 (52.9)		0.465
MBL levels ≤700 ng/mL	68 (45.3)		72 (53.7)		0.411	44 (53.0)		28 (54.9)		0.907

^*^Statistical significance *P*1 and *P*2 refer to comparison of *MBL2* genotypes and MBL concentrations between NICU patients with healthy controls (chi-square and Mann-Whitney *U* test, resp.); ^#^ *P*3 and *P*4 refer to comparison of *MBL2* genotypes and MBL concentrations between term and preterm neonates of NICU (chi-square and Mann-Whitney *U* test, resp.);  ^∧^ *P*5 and *P*6 refer to comparison of MBL functional deficiency (using 3 different cut-offs) between healthy and NICU neonates and between term and preterm neonates of NICU, respectively (chi-square test).

**Table 3:** Overview of *MBL2* genotypes and haplotypes in the subjects of the study.

	NICU (neonatal intensive care)					
	Healthy (*n* 150)	Total (*n* 134)		Term (*n* 83)	Preterm (*n* 51)	
	*n* (%)	*n* (%)	*P*1^*^	*n* (%)	*n* (%)	*P*2^*^
*MBL2 *genotypes						
YA/YA						
HYA/HYA	13 (8.7)	10 (7.5)	0.732	6 (7.2)	4 (7.8)	0.903
LYA/LYA	7 (4.7)	4 (3.0)	0.480	1 (1.2)	3 (5.9)	0.136
HYA/LYA	31 (20.7)	28 (20.9)	0.969	16 (19.3)	12 (23.5)	0.636

Y*Α*/Χ*Α*						
LYA/LXA	9 (6.0)	10 (7.5)	0.645	4 (4.8)	6 (11.8)	0.171
HYA/LXA	16 (10.7)	11 (8.2)	0.521	9 (10.8)	2 (3.9)	0.188

Χ*Α*/Χ*Α*						
LXA/LXA	6 (4.0)	9 (6.7)	0.333	7 (8.4)	2 (3.9)	0.341

Y*Α*/O						
HYA/HYD	4 (2.7)	2 (1.5)	0.501	2 (2.4)	0 (0)	0.270
HYA/LYB	26 (17.3)	20 (14.9)	0.640	15 (18.1)	5 (9.8)	0.257
HYA/LYC	0 (0)	1 (0.7)	0.291	1 (1.2)	0 (0)	0.434
LYA/HYD	4 (2.7)	6 (4.5)	0.425	3 (3.6)	3 (5.9)	0.557
LYA/LYB	8 (5.3)	4 (3.0)	0.346	3 (3.6)	1 (2.0)	0.595

XA/O						
LXA/LYB	9 (6.0)	14 (10.4)	0.206	9 (10.8)	5 (9.8)	0.863
LXA/HYD	7 (4.7)	2 (1.5)	0.139	1 (1.2)	1 (2.0)	0.730
LXA/LYC	0 (0)	1 (0.7)	0.291	1 (1.2)	0 (0)	0.434

O/O						
LYB/LYB	5 (3.3)	7 (5.2)	0.449	4 (4.8)	3 (5.9)	0.799
LYB/HYD	5 (3.3)	3 (2.2)	0.588	1 (1.2)	2 (3.9)	0.314
HYD/HYD	0 (0)	2 (1.5)	0.136	0 (0)	2 (3.9)	0.075

*MBL2 *haplotypes						
HYA	103 (34.3)	82 (30.6)	0.498	55 (33.1)	27 (26.5)	0.399
LYA	66 (22.0)	56 (20.9)	0.797	28 (18.9)	28 (27.5)	0.097
LXA	53 (17.7)	56 (20.9)	0.422	38 (22.9)	18 (17.6)	0.404
LYB	58 (19.3)	55 (20.5)	0.772	36 (21.7)	19 (18.6)	0.624
HYD	20 (6.7)	17 (6.3)	0.884	7 (4.2)	10 (9.8)	0.089
LYC	0	2 (0.7)	0.135	2 (1.2)	0 (0)	0.269

^*^Statistical significance (chi-square test) *P*1 refers to comparison of *MBL2* genotypes between NICU and healthy neonates and *P*2 to comparison of *MBL2* genotypes between term and preterm neonates of NICU.

## References

[1] Strunk T., Burgner D. (2006). Genetic susceptibility to neonatal infection. *Current Opinion in Infectious Diseases*.

[2] Levy O. (2007). Innate immunity of the newborn: basic mechanisms and clinical correlates. *Nature Reviews Immunology*.

[3] Hedberg C. L., Adcock K., Martin J., Loggins J., Kruger T. E., Baier R. J. (2004). Tumor necrosis factor *α*—308 polymorphism associated with increased sepsis mortality in ventilated very low birth weight infants. *The Pediatric Infectious Disease Journal*.

[4] Harding D., Dhamrait S., Millar A. (2003). Is interleukin-6 -174 genotype associated with the development of septicemia in preterm infants?. *Pediatrics*.

[5] Frakking F. N. J., Brouwer N., Zweers D. (2006). High prevalence of mannose-binding lectin (MBL) deficiency in premature neonates. *Clinical and Experimental Immunology*.

[6] Bodamer O. A., Mitterer G., Maurer W., Pollak A., Mueller M. W., Schmidt W. M. (2006). Evidence for an association between mannose-binding lectin 2 (MBL2) gene polymorphisms and pre-term birth. *Genetics in Medicine*.

[7] van der Zwet W. C., Catsburg A., van Elburg R. M., Savelkoul P. H. M., Vandenbroucke-grauls C. M. J. E. (2008). Mannose-binding lectin (MBL) genotype in relation to risk of nosocomial infection in pre-term neonates in the neonatal intensive care unit. *Clinical Microbiology and Infection*.

[8] St. Swierzko A., Atkinson A. P. M., Cedzynski M. (2009). Two factors of the lectin pathway of complement, l-ficolin and mannan-binding lectin, and their associations with prematurity, low birthweight and infections in a large cohort of Polish neonates. *Molecular Immunology*.

[9] Hilgendorff A., Heidinger K., Pfeiffer A. (2007). Association of polymorphisms in the mannose-binding lectin gene and pulmonary morbidity in preterm infants. *Genes and Immunity*.

[10] de Benedetti F., Auriti C., D'Urbano L. E. (2007). Low serum levels of mannose binding lectin are a risk factor for neonatal sepsis. *Pediatric Research*.

[11] Ahrens P., Kattner E., Köhler B. (2004). Mutations of genes involved in the innate immune system as predictors of sepsis in very low birth weight infants. *Pediatric Research*.

[12] Frakking F. N. J., Brouwer N., van Eijkelenburg N. K. A. (2007). Low mannose-binding lectin (MBL) levels in neonates with pneumonia and sepsis. *Clinical and Experimental Immunology*.

[13] Turner M. W. (2003). The role of mannose-binding lectin in health and disease. *Molecular Immunology*.

[14] Thiel S., Vorup-Jensen T., Stover C. M. (1997). A second serine protease associated with mannan-binding lectin that activates complement. *Nature*.

[15] Tenner A. J., Robinson S. L., Ezekowitz R. A. B. (1995). Mannose binding protein (MBP) enhances mononuclear phagocyte function via a receptor that contains the 126,000 M_r_ component of the C1q receptor. *Immunity*.

[16] Ghiran I., Barbashov S. F., Klickstein L. B., Tas S. W., Jensenius J. C., Nicholson-Weller A. (2000). Complement receptor 1/CD35 is a receptor for mannan-binding lectin. *The Journal of Experimental Medicine*.

[17] Sallenbach S., Thiel S., Aebi C. (2011). Serum concentrations of lectin-pathway components in healthy neonates, childrens and adults: mannan-binding lectin (MBL), M-, L-, and H-ficolin, and MBL-associated serine protease-2 (MASP-2). *Pediatric Allergy and Immunology*.

[18] Garred P., Larsen F., Seyfarth J., Fujita R., Madsen H. O. (2006). Mannose-binding lectin and its genetic variants. *Genes and Immunity*.

[19] Madsen H. O., Garred P., Thiel S. (1995). Interplay between promoter and structural gene variants control basal serum level of mannan-binding protein. *Journal of Immunology*.

[20] Koch A., Melbye M., Sørensen P. (2001). Acute respiratory tract infections and mannose-binding lectin insufficiency during early childhood. *The Journal of the American Medical Association*.

[21] Summerfield J. A., Sumiya M., Levin M., Turner M. W. (1997). Association of mutations in mannose binding protein gene with childhood infection in consecutive hospital series. *British Medical Journal*.

[22] Neth O. W., Bacher U., Das P. (2010). Influence of mannose-binding lectin genotypes and serostatus in allo-SCT: analysis of 131 recipients and donors. *Bone Marrow Transplantation*.

[23] Fioretto J. R., de Carvalho W. B. (2013). Temporal evolution of acute respiratory distress syndrome definitions. *Jornal de Pediatria*.

[24] Tin S. K., Lee L. Y., Thumboo J., Koh D. R., Fong K. Y. (2005). PCR-RFLP genotyping for exon 1 and promoter region mutations of the human mannose binding lectin (MBL-2) gene. *Journal of Immunological Methods*.

[25] Aittoniemi J., Miettinen A., Laippala P. (1996). Age-dependent variation in the serum concentration of mannan-binding protein. *Acta Paediatrica, International Journal of Paediatrics*.

[26] Chroneos Z. C., Sever-Chroneos Z., Shepherd V. L. (2010). Pulmonary surfactant: an immunological perspective. *Cellular Physiology and Biochemistry*.

[27] Halliday H. L. (2008). Surfactants: past, present and future. *Journal of Perinatology*.

[28] Sweet D. G., Carnielli V., Greisen G. (2010). European consensus guidelines on the management of neonatal respiratory distress syndrome in preterm infants—2010 update. *Neonatology*.

[29] Gong M. N., Zhou W., Williams P. L., Thompson B. T., Pothier L., Christiani D. C. (2007). Polymorphisms in the mannose binding lectin-2 gene and acute respiratory distress syndrome. *Critical Care Medicine*.

[30] Lin C. L., Siu L. K., Lin J. C. (2011). Mannose-binding lectin gene polymorphism contributes to recurrence of infective exacerbation in patients with COPD. *Chest*.

[31] Chalmers J. D., Fleming G. B., Hill A. T., Kilpatrick D. C. (2011). Impact of mannose-binding lectin insufficiency on the course of cystic fibrosis: a review and meta-analysis. *Glycobiology*.

[32] Fevang B., Mollnes T. E., Holm A. M. (2005). Common variable immunodeficiency and the complement system; low mannose-binding lectin levels are associated with bronchiectasis. *Clinical & Experimental Immunology*.

[33] Litzman J., Freiberger T., Grimbacher B. (2008). *Mannose-binding lectin* gene polymorphic variants predispose to the development of bronchopulmonary complications but have no influence on other clinical and laboratory symptoms or signs of common variable immunodeficiency. *Clinical and Experimental Immunology*.

[34] Chalmers J. D., McHugh B. J., Doherty C. (2013). Mannose-binding lectin deficiency and disease severity in non-cystic fibrosis bronchiectasis: a prospective study. *The Lancet Respiratory Medicine*.

[35] Garred P., Pressler T., Madsen H. O. (1999). Association of mannose-binding lectin gene heterogeneity with severity of lung disease and survival in cystic fibrosis. *The Journal of Clinical Investigation*.

[36] Garred P., Pressler T., Lanng S. (2002). Mannose-binding lectin (MBL) therapy in an MBL-deficient patient with severe cystic fibrosis lung disease. *Pediatric Pulmonology*.

[37] Seaton B. A., Crouch E. C., McCormack F. X., Head J. F., Hartshorn K. L., Mendelsohn R. (2010). Structural determinants of pattern recognition by lung collectins. *Innate Immunity*.

[38] Eggleton P., Reid K. B. (1999). Lung surfactant proteins involved in innate immunity. *Current Opinion in Immunology*.

[39] Duus K., Thielens N. M., Lacroix M. (2010). CD91 interacts with mannan-binding lectin (MBL) through the MBL-associated serine protease-binding site. *FEBS Journal*.

[40] Palaniyar N., Nadesalingam J., Clark H., Shih M. J., Dodds A. W., Reid K. B. M. (2004). Nucleic acid is a novel ligand for innate, immune pattern recognition collectins surfactant proteins A and D and mannose-binding lectin. *The Journal of Biological Chemistry*.

