# Supplementary figures and images for: MBL2 Genotypes and Their Associations with MBL Levels and NICU Morbidity in a Cohort of Greek Neonates
Source: J Immunol Res. 2015 Mar 24;2015:478412. doi: 10.1155/2015/478412 (PMC4387979; doi:10.1155/2015/478412)

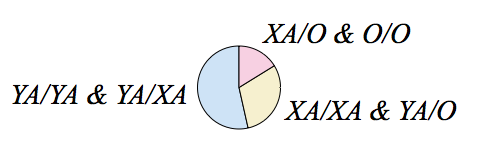

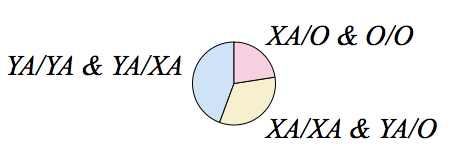

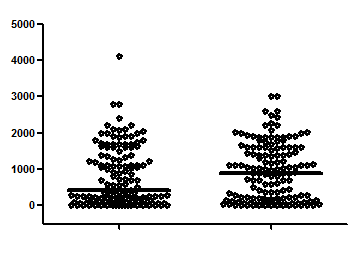


23/142 (16.2%)

43/142 (30.3%)

76/142 (53.5%)

**FEMALE**

47/142 (33.1%)

32/142 (22.5%)

63/142 (44.4%)

**MALE**

**B.**

**MBL concentration (ng/mL)**

**A.**

**Male Female**

***p* = 0.095**

Supplement: Supplementary file 1 — Supplementary Figure 1. A. Scatterplot of MBL serum levels and B. prevalence of MBL2 genotype groups in the neonates of the study, according to their sex. The graph B was prepared by “Mac Statistics Wizard” software (version 10.0.4) ". Supplementary Table 1. Clinical and demographic characteristics of the study population. [file 478412.f1.docx]
