# Supplementary material for: MBL2 Genotypes and Their Associations with MBL Levels and NICU Morbidity in a Cohort of Greek Neonates
Source: J Immunol Res. 2015 Mar 24;2015:478412. doi: 10.1155/2015/478412 (PMC4387979; doi:10.1155/2015/478412)
Supplement: Supplementary file 2 [file 478412.f2.docx]

**Supplementary Table 1**. Clinical and demographic characteristics of the study population.

|  | **Healthy** | **NICU** | | |
| --- | --- | --- | --- | --- |
|  | (n 150) | **Total**  (n 134) | **Term**  (n 83) | **Preterm**  (n 51) |
| ***Neonate characteristics*** | | | | |
| Gender, male (n, %) | 62 (41.3) | 80 (59.7) | 49 (59.0) | 31 (60.8) |
| Birth weight (gr)  (median, range) | 3275  (2200-4350) | 2790  (880-5050) | 3080  (1610-5050) | 2020  (880-3400) |
| Gestational age (weeks)  (median, range) | 40 (36-41) | 37 (27-41) | 39 (37-41) | 35 (27-36) |
| Prematurity (<37 weeks)  (no, %) | 2 (1.3) | 51 (38.1) | 0 (0) | 51 (100.0) |
| SGA (n, %) | 4 (2.7) | 21 (15.7) | 17 (20.5) | 4 (7.8) |
| LGA (n, %) | 1 (0.7) | 6 (4.5) | 5 (6.0) | 1 (2.0) |
| Caesarian section (n, %) | 46 (30.7) | 81 (60.4) | 45 (54.2) | 36 (70.6) |
| ***Pregnancy-related disease*** | | | | |
| Diabetes gravidarum (n, %) | 2 (1.3) | 13 (9.7) | 9 (10.8) | 4 (7.8) |
| Hypertension gravidarum (n, %) | 3 (2.0) | 3 (2.2) | 1 (1.2) | 2 (3.9) |
| Preeclampsia (n, %) | 1 (0.7) | 2 (1.5) | 1 (1.2) | 1 (2.0) |
| Chorioamnionitis (n, %) | 2 (1.3) | 14 (10.4) | 8 (9.6) | 5 (9.8) |
| ***Condition of neonates*** | | | | |
| Respiratory morbidity (n, %) | 0 (0) | 80 (59.7) | 44 (53.0) | 36 (70.6) |
| RDS (n, %) | 0 (0) | 27 (20.1) | 8 (9.6) | 19 (37.3) |
| TTN (n, %) | 0 (0) | 28 (20.9) | 13 (15.7) | 15 (29.4) |
| Perinatal asphyxia (n, %) | 0 (0) | 23 (17.2) | 21 (25.3) | 2 (3.9) |
| Airleaks (n, %) | 0 (0) | 9 (6.7) | 5 (6.0) | 4 (7.8) |
| PPHN (n, %) | 0 (0) | 2 (1.5) | 1 (1.2) | 1 (2.0) |
| Congenital pneumonia (n,%) | 0 (0) | 2 (1.5) | 2 (2.4) | 0 (0) |
| Seizures (n, %) | 0 (0) | 5 (3,7) | 4 (4.8) | 1 (2.0) |
| Perinatal stress (n, %) | 0 (0) | 18 (13.4) | 15 (18.1) | 3 (5.9) |
| Jaundice (n, %) | 3 (2.0) | 51 (38.1) | 22 (26.5) | 29 (56.9) |
| Perinatal infections (n, %) | 0 (0) | 19 (14.2) | 12 (14.5) | 7 (13.7) |
| Gram (+) (n, %) | 0 (0) | 9 (6.7) | 5 (6.0) | 4 (7.8) |
| Gram (-) (n, %) | 0 (0) | 2 (1.5) | 0 (0) | 2 (3.9) |
| Sepsis (culture proven) (n, %) | 0 (0) | 11 (8.2) | 5 (6.0) | 6 (11.8) |
| Death | 0 (0) | 2 (1.5) | 1 (1.2) | 1 (2.0) |

**Abbreviations**: NICU, neonate intensive care unit; LGA, large for gestational age; RDS, respiratory distress syndrome; PPHN, persistent pulmonary hypertension of the newborn; SGA: small for gestational age; TTN, transient tachypnea of the newborn
